# Development of PowerMag System II for Isolation of Circulating Tumor Cells with Improved Purity

**DOI:** 10.3390/biomedicines13020431

**Published:** 2025-02-11

**Authors:** Cheng-Rou Ho, Hui-Ju Tsai, Jin-Ru Wang, Chia-Te Wang, Chiuan-Chian Chiou, Ju-Chien Cheng, Sum-Fu Chiang, Ching-Ping Tseng

**Affiliations:** 1 Department of Medical Biotechnology and Laboratory Science, College of Medicine, Chang Gung University, Taoyuan 333, Taiwan; 2 International Master Degree Program for Molecular Medicine in Emerging Viral Infections, College of Medicine, Chang Gung University, Taoyuan 333, Taiwan; 3 Master and PhD Program in Biotechnology Industry, College of Medicine, Chang Gung University, Taoyuan 333, Taiwan; 4 Department of Thoracic Medicine, Chang Gung Memorial Hospital, Linkou Branch, Taoyuan 333, Taiwan; 5 Department of Medical Laboratory Science and Biotechnology, China Medical University, Taichung 404, Taiwan; 6 Division of Colon and Rectal Surgery, Chang Gung Memorial Hospital, Linkou Branch, Taoyuan 333, Taiwan; 7 School of Traditional Chinese Medicine, Chang Gung University, Taoyuan 333, Taiwan; 8 Graduate Institute of Biomedical Science, College of Medicine, Chang Gung University, Taoyuan 333, Taiwan; 9 Department of Laboratory Medicine, Chang Gung Memorial Hospital, Linkou Branch, Taoyuan 333, Taiwan

**Keywords:** annexin V, apoptosis, cancer, circulating tumor cells

## Abstract

**Background/Objectives**: The PowerMag system (PM) is a platform for the isolation of circulating tumor cells (CTCs) by the depletion of CD45^+^-leukocytes. However, an EpCAM^−^CD45^−^ cell population is present in large numbers in the cell filtrates collected by PM. This lowers the purity of the CTCs and negatively impacts their molecular characterization. The aims of this study are to characterize the cellular properties of the EpCAM^−^CD45^−^ cells and to upgrade the system to improve CTC purity. **Methods**: A real-time RT-PCR assay, Liu’s stain analysis, and Annexin V (AnxV) binding assay were used to define the cellular properties of the EpCAM^−^CD45^−^ cells. An upgraded system was developed to remove the EpCAM^−^CD45^−^ cells and improve the CTC purity. Clinical blood samples were used to evaluate the performance of the system. **Results**: The EpCAM^−^CD45^−^ cells were defined as apoptotic cells, which displayed apoptotic body-like morphology and elicited AnxV binding activity. AnxV beads developed in-house can effectively bind and remove EpCAM^−^CD45^−^ cells from the cell filtrates. An improved generation of a CTCs isolation platform, designated as PM II, was developed by integration of AnxV beads into the workflow to remove the apoptotic cells. PM II recovered CTCs with improved CTC purity by effective removal of the background apoptotic cells. The improved performance of PM II allowed for direct profiling of cancer-related gene mutations by next-generation sequencing without cell picking and further purification. **Conclusions**: PM II holds great promise as a platform for isolating CTCs with improved purity and for exploring its application in cancer diagnosis and monitoring in a clinical setting.

## 1. Introduction

Cancer cells are released into the peripheral blood to form circulating tumor cells (CTCs) during cancer progression [1]. The number of CTCs in the blood stream is associated with cancer metastasis and relapse [2,3,4]. It is a type of cancer liquid biopsy that can be used for disease detection and monitoring, new drug development, and the selection of therapeutic regimens [5,6,7]. CTC enumeration thereby provides crucial information for patient care in a clinical setting.

Various strategies have been developed for the enrichment and enumeration of CTCs from peripheral blood [8,9,10]. There are many methods that are based on either the physical or biological properties of blood cells and cancer cells [11,12,13,14,15,16], including several technical platforms that either operate manually or automatically [13,16,17,18]. We have developed a PowerMag (PM) system for the enrichment and enumeration of CTCs, which is mainly operated by a negative selection mode [16]. Peripheral blood is subject to RBC lysis, depletion of CD45^+^-leukocytes through a PM magnetic column, immunostaining of the cell filtrates with the antibodies against the epithelial cell marker (epithelial cell adhesion molecule, EpCAM), and the leukocyte marker (CD45), followed by enumeration of the CTCs (EpCAM^+^CD45^−^) through fluorescence microscopy. Theoretically, all types of CTCs, which are either epithelial marker positive or negative, can be fully recovered from peripheral blood by this method. Negative selection and enrichment of CTCs by the PM, therefore, represents a better approach for CTC isolation and enumeration. This platform has been used successfully in disease monitoring and evaluation of treatment efficacy for oral cancer, thyroid cancer, hepatocellular carcinoma, and colorectal cancer [19,20,21,22,23,24,25,26].

In addition to EpCAM^+^CD45^−^-CTCs, a cell population that was negative for both epithelial cells and leukocyte markers (i.e., EpCAM^−^CD45^−^) is present in the cell filtrates after leukocyte depletion by PM [16]. This has been observed with CTC isolation platforms based on negative selection [27,28]. There are EpCAM^−^CD45^−^ cells in healthy donors, as well as cancer patients, with the number usually higher in cancer patients when compared to the healthy controls [16]. The higher number of this cell population in the cell filtrates significantly hampers the purity of the CTCs and has a negative impact on further molecular characterization, such as the analysis of genetic mutations in the CTCs by next-generation sequencing (NGS).

There is an unmet need to investigate the cellular properties of EpCAM^−^CD45^−^ cells and to further improve the purity of CTCs isolated by the PM. In this study, we unveiled that EpCAM^−^CD45^−^ cells mainly represent the apoptotic cells in the cell filtrate collected by PM. Based on the above information, a new generation of the PM designated as PM II was developed by the integration of in-house Annexin V (AnxV) beads into the workflow of the original version of the PM, designated as PM I, to remove the apoptotic cells. PM II improved CTC purity, indicating its suitability for the direct molecular characterization of CTC genetic mutations by NGS.

## 2. Materials and Methods

### 2.1. Materials

TaqMan probe and real-time RT-PCR reagents were purchased from Thermo (Waltham, MA, USA). The fluorescein isothiocyanate (FITC)-conjugated anti-CD45 antibody and the phycoerythrin (PE)-conjugated antibodies against CD11b, CD14, and CD19 were purchased from eBioscience (San Diego, CA, USA). The PE-labeled anti-EpCAM antibody was purchased from BioLegend (San Diego, CA, USA). The anti-CD45 depletion kit was purchased from StemCell Technologies (Vancouver, BC, Canada). Cytocentrifuge (Cytopro 7622) was purchased from ELITechGroup (Puteaux, Paris, France). The His-tag AnxV plasmid (pET-14b-AnxV) has been described previously [29]. The 1× AnxV binding buffer and AnxV conjugated with FITC (AnxV-FITC) were purchased from Abcam (Cambridge, MA, USA). The magnetic nickel beads were purchased from Promega (Madison, WI, USA). The anti-histidine antibody was purchased from Bio-Rad (Hercules, CA, USA). The QIAamp DNA mini kit, REPLI-g mini kit, and QIAseq target DNA pro colorectal cancer research panel were purchased from Qiagen (Hulsterweg, Venlo, The Netherlands). All other chemicals were purchased from SIGMA (Darmstadt, Hesse, Germany).

### 2.2. Blood Collection

This study was approved by the Institute Review Board of Chang Gung Memorial Hospital, with the approval IDs of 201801888B0 and 201601848B0. The peripheral blood (4 mL/test) was drawn from healthy donors and colorectal cancer patients with written informed consent and was collected in a blood collection tube containing EDTA. The blood samples were maintained at 4 °C until processing by PM.

### 2.3. TaqMan Real-Time RT-PCR Assay

The cell suspension (1 μL) was mixed with 5 μL of 2× reaction mix and 0.1 μL of SUPERase inhibitor in a PCR tube. Cells were lysed, and cellular RNA was released by vortexing the mixture for 15 sec. Taq DNA polymerase (0.2 μL) and nuclease-free water (2.7 μL) were added to the mixture, with a final volume of 10 μL. Reverse transcription was performed at 55 °C for 10 min to convert RNAs into cDNAs. Subsequently, 4 μL cDNAs were mixed with 10 μL 2× master mix, 1 μL 20× TaqMan probe, and 5 μL nuclease-free water to obtain the PCR assay mixture (20 μL). Real-time PCR was performed by using the LightCycler 480 (Roche, Basel, Switzerland) with the following steps: incubation at 50 °C for 2 min, initial denaturation at 95 °C for 10 min, followed by denaturation at 95 °C for 15 s, and annealing/extension at 60 °C for 1 min, for a total of 40 cycles.

The performance of the TaqMan probe and real-time RT-PCR assay was evaluated by using the following cell types as the positive control. White blood cells collected from the buffy coat of the peripheral blood were used as a positive control for the real-time RT-PCR assay of CD11b and CD14. The peripheral blood mononuclear cell (PBMC) collected from the Ficoll-Paque density gradient of peripheral blood was used as a positive control for the real-time RT-PCR assay of CD19 and CD2. The granulocytes collected from the Ficoll-Paque density gradient of peripheral blood were used as a positive control for real-time RT-PCR assay of CD66b, CD45, and CD16. Ficoll-Paque density gradient centrifugation of peripheral blood was performed as described previously [30]. NB4 and HEK293 cells [31,32] were used as the positive control for the real-time RT-PCR assay of CD11c and CD56, respectively. In addition, no template control (NTC) was used as the negative control of the assay, while real-time RT-PCR using the β-actin TaqMan probe was used as a control for the quality of sample preparation.

### 2.4. Preparation of Magnetic Beads Conjugated with Annexin V Protein (AnxV Beads)

The His-tag AnxV plasmid (pET-14b-AnxV) was transformed into BL21 competent cells. The expression of His-AnxV protein was induced by incubation of the transformed bacteria with IPTG. His-AnxV protein (0.5 μg/μL) in protein purification buffer (100 mM HEPES, 20 mM imidazole, 300 mM NaCl, 10 mM β-mercaptoethanol, and 1 mM PMSF) was mixed with the magnetic nickel beads in a 20:3 ratio and incubated at room temperature for 2 h. The supernatant was discarded, and the beads were washed twice with wash buffer (100 mM HEPES, 50 mM imidazole, and 300 mM NaCl). The AnxV beads were stored in the storage buffer (1× phosphate-buffered saline (PBS) with 0.02% NaN_3_, 1% BSA, and 1 mM PMSF) at 4 °C until use.

### 2.5. Western Blot Analysis and Coomassie Blue Staining

Protein lysates were fractionated on a 12% sodium dodecyl sulfate–polyacrylamide gel by electrophoresis for 3 h with a running voltage of 80 V. The fractionated proteins were transferred onto the polyvinylidene difluoride membrane and incubated with 5% dry milk for 1 h to block the non-specific binding sites of the membrane. After incubation with the mouse anti-histidine antibody (1:20,000) for 1 h, the membrane was washed three times using 1× PBS containing 0.1% Tween 20 (0.1% PBST). The horseradish peroxidase (HRP)-conjugated goat anti-mouse antibody (1:20,000) was then applied for 30 min. After washing three times with 0.1% PBST, a mixture of luminol and peroxide was added to the membrane and incubated for 1 min. Autoradiography was performed to visualize the luminescence signal on film.

For Coomassie blue staining, the polyacrylamide gel was gently fixed in fixation buffer (50% methanol and 25% acetic acid) for 1 h. After staining overnight with Coomassie blue, the excessive dye was washed off, and gel imaging was performed.

### 2.6. Cell Culture

The Jurkat and SW480 cell lines were purchased from the American Type Culture Collection (Manassas, VA, USA). The OECM-1 cell line was obtained from Professor Ann-Joy Cheng (Department of Medical Biotechnology and Laboratory Science, College of Medicine, Chang Gung University, Taoyuan, Taiwan). Jurkat cells and SW480 cells were cultured in Roswell Park Memorial Institute (RPMI) medium supplemented with 10% fetal bovine serum (FBS). OECM-1 cells were cultured in RPMI medium supplemented with 5% FBS. All media were supplemented with 1% penicillin/streptomycin and were filtered using a 0.2 μm filter before use.

### 2.7. Staurosporine-Induced Apoptosis of Jurkat Cells

For the induction of apoptosis, Jurkat cells were suspended in serum-free RPMI and seeded (2 × 10^6^ cells) in a 12-well plate. Staurosporine (1 μg/mL) was then added to the plate for incubation at 37 °C for 3 h. Apoptotic cells were defined by the observation of cell morphology and binding of AnxV-FITC. For the analysis of cell morphology, cells were collected and applied onto slides via cytospin for Liu’s stain (Liu A for 40 s and Liu B for 75 s), followed by microscopy analysis using the optical microscope (ZEISS Primostar 1, ZEISS, Oberkochen, Germany).

For fluorescent staining, 100 μL of cell solution was centrifuged, and the medium was replaced with 100 μL of 1× AnxV binding buffer. To this, 2 μL of AnxV-FITC and 2 μL of Hoechst 33342 were added. After 5 min in the dark at room temperature and a wash, cells were resuspended in a 10% RPMI medium for microscopy.

### 2.8. Flow Cytometry Analysis

A cell suspension containing 2 × 10^5^ Jurkat cells in 100 μL of 1× AnxV-binding buffer was incubated with 2 μL of AnxV-FITC and 2 μL of propidium iodide staining dye at room temperature for 5 min according to the instructions of the manufacturer (Abcam). The cell suspension was then washed once with the culture medium. The cell pellet was obtained by centrifugation at 600× *g* for 3 min and was resuspended in 500 μL of cell culture medium containing 5 mM CaCl_2_. For the flow cytometry analysis, cells with a forward scatter (FSC) < 500,000 were excluded, and a total of 20,000 cells were captured for analysis.

### 2.9. PM I and PM II for CTCs Enrichment

**Figure 1:**
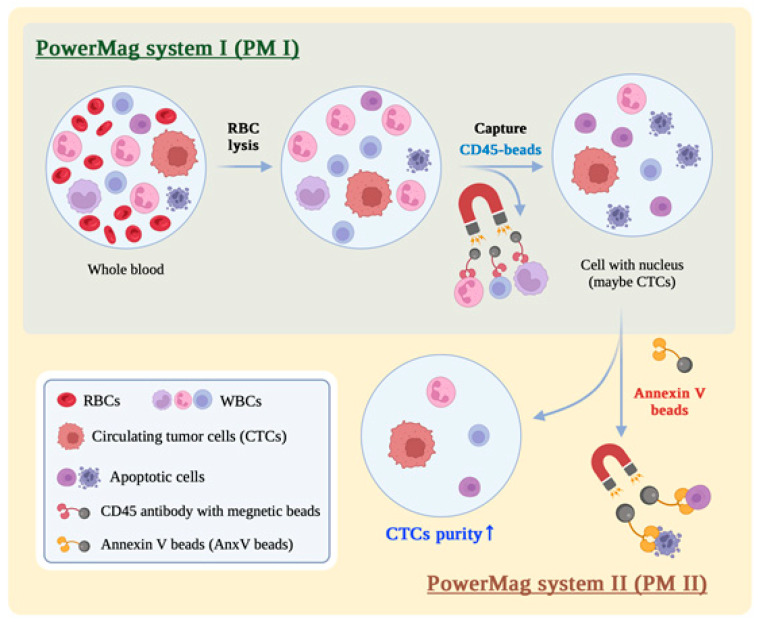
Schematic representation for the workflow of PM I and PM II. PM II was developed by integrating and placing AnxV beads after the workflow of PM I for removal of apoptotic cells.

The PM system, which includes a custom-made magnetic chamber and a bead-packed plastic separation column (PM column), has been described previously for the negative selection of CTCs [16,26]. The workflow for CTC enrichment using the PM system was designated as PM I in this study (Figure 1). Briefly, whole blood (2 mL) was mixed with RBC lysis buffer (0.15 M NH_4_Cl and 10 mM NaHCO_3_) for 5 min at room temperature to lyse the erythrocytes. After centrifuging the mixture (400× *g*) at 10 °C for 10 min, cell pellets containing the nucleated cells were washed and resuspended in a cell culture medium (4 mL). For the depletion of CD45^+^ leukocytes, the collected nucleated cells were mixed with the CD45 depletion cocktail at room temperature for 5 min, followed by adding the dextran-coated magnetic nanoparticles to the reaction mixture and incubating at room temperature for 3 min. To separate the CD45^+^-leukocytes from the other nucleated cells, the reaction mixture was loaded into the PM column to capture CD45^+^-leukocytes on the surface of the separation beads. The cell filtrates containing CD45^−^ cells were centrifuged (800× *g*) for 6 min at 10 °C. The pelleted cells were collected and mixed with the CD45 depletion cocktail to repeat the depletion process a total of 3 times.

The system and the workflow for CTC enrichment were designated as PM II when AnxV beads were integrated into PM I for the depletion of apoptotic cells (Figure 1). Briefly, the cell pellets obtained from the final step of PM I were resuspended in 500 μL of cell culture medium containing 5 mM CaCl_2_. The AnxV beads (2 μg of AnxV protein in 10 μL) were then added into the cell suspension and incubated for 20 min at room temperature. During the process, apoptotic cells were bound on the surface of the AnxV beads. The mixture of cells and beads was captured on the interior side of eppendorf tube by using the magnetic stand, allowing for the capture of AnxV beads and the retention of apoptotic cells. The cell filtrate was then centrifuged (600× *g*) for 3 min at 10 °C. The cell pellets were resuspended in 10 μL of cell culture medium for further analysis.

### 2.10. Immunofluorescence Staining for CTC Enumeration

For the enumeration of CTCs by immunofluorescence staining, anti-EpCAM antibody and the Hoechst 33342 DNA staining dye were mixed with the cell filtrates obtained by the PM system and incubated at room temperature for 1 h. After centrifugation and washing twice, Alexa Fluor 594-conjugated goat anti-rabbit antibody was added to the cell suspension and kept in the dark for 30 min. On some occasions, FITC-labeled anti-CD45 antibody was added to the cell mixture to stain CD45^+^ cells and differentiate CD45^+^-leukocytes from other nucleated cells. After the removal of the unbound antibody, the cell filtrates were loaded onto a 6-channel μ-Slide. The numbers of fluorescence cells were counted by automated scanning of the channel using fluorescence microscopy (Zeiss Axiovert 200M, Oberkochen, Germany).

### 2.11. Analysis of CTCs Detection Limit

The SW480 cells were pre-labeled with the fluorescence Calcein Red™ AM viability dye. The cells were diluted to 1 cell/10 μL and 10 cells/10 μL. An aliquot of the cell suspension was loaded onto a 6 channel μ-Slide (Ibidi, Gräfelfing, Germany). The cell number was confirmed by counting the cells in the channel using the fluorescence microscope with an automated stage to scan the whole channel. Then, the fluorescence-labeled SW480 cells (1, 10, or 1000 cells) were spiked into the peripheral blood obtained from healthy donors to mimic clinical samples from cancer patients. After processing through the workflow of PM I and PM II, the cells were resuspended in 10 μL of cell culture medium and loaded onto a 6 channel μ-Slide for counting the number of fluorescence cells by automated scanning of the channel using fluorescence microscopy.

### 2.12. Genomic DNA Isolation

Genomic DNA (gDNA) was isolated by using the QIAamp DNA mini Kit according to the instructions of the manufacturer (Qiagen). Briefly, the cells were washed once using 1 mL of 1× PBS. After centrifugation, the supernatant was removed, and the cell pellet was resuspended in 200 μL of 1× PBS containing protease K and RNase A. Buffer AL was added to the mixture for incubation at 56 °C for 10 min. Subsequently, absolute ethanol was added to the mixture, and the solution was transferred to a QIAamp mini spin column, along with a collection tube, allowing the gDNA to bind to the column membrane. The waste in the collection tube was discarded, and the column was washed using Buffer AW1 and AW2. After centrifugation at the highest speed for 5 min to dry the membrane, elution was performed using 100 μL of ddH_2_O. The gDNA was then quantified using a nanodrop and Qubit and applied to a concentrator for 1 h to condense the DNA concentration if required.

### 2.13. Whole-Genome Amplification (WGA)

Whole-genome amplification (WGA) was performed by using the REPLI-g mini kit according to the instructions of the manufacturer (Qiagen). Briefly, the gDNA was resuspended in 5 μL of Tris-HCl (pH 8.5) and subject to WGA by adding 5 μL of Buffer D1 for the denaturation of the DNA. After incubating at room temperature for 3 min, 10 μL of Buffer N1 were added for neutralization. A WGA master mix containing random primers and phi29 DNA polymerase was prepared, and the denatured gDNA was added to the master mix. WGA was performed at 30 °C for 16–18 h. The reaction was stopped by incubating the assay mixture at 65 °C for 3 min. The DNA concentration was quantified using a NanoDrop and Qubit, and agarose gel electrophoresis was conducted for further analysis. The amplified DNA was then used for NGS sequencing.

### 2.14. NGS Sequencing and Bioinformatics Analysis

NGS was performed by Genomics Inc. (Xizhi, New Taipei, Taiwan). Briefly, the amplified gDNA was quantified using a NanoDrop and Qubit dsDNA HS assay kit. Qualitative analysis was performed using a Fragment Analyzer 5200 and a DNF-930 kit. After library preparation, the DNA was sequenced on an Illumina NovaSeq^TM^ 6000 using a QIAseq-targeted DNA pro human colorectal cancer research panel (#PHS-002Z). The resulting FASTQ file was processed with smCounter2 [33] to remove low-quality reads, followed by alignment to the human genome 38 (hg38). The data were saved as a BAM file. Information on the chromosome location and position was then exported by SAMtools [34] and saved as a BED file. BEDTools [35] was employed to consolidate repeat data, minimizing the size of the BED file. Subsequently, the BAM-readcount [36] tool was utilized to identify the counts of A/T/C/G within these specified regions with the quality criteria set at base quality (BQ) = 30 and mapping quality (MQ) = 60. Finally, BEDtools was used for mapping the regions outlined in the colorectal cancer panel, and the consolidated data was exported as a txt file. Following this, Google Colaboratory [37] was employed for variants filtering to refine the dataset. Specifically, positions with total reads of less than 5000 were filtered out. Positions exhibiting mutant read counts of more than 100 were retained for analysis. Finally, the mutation information underwent annotation via the wANNOVAR website [38], leveraging multiple databases including dbSNP [39], COSMIC [40], ClinVar [41], and others. dbSNP is the most comprehensive database for cataloging short variations, including single nucleotide variations, as well as insertions, deletions, and short tandem repeats in the human genome. The COSMIC and ClinVar databases were selected for variant annotations because both databases provide the most comprehensive resource for exploring the impact of somatic mutations in human cancer or oncogenicity.

### 2.15. Statistical Analysis

Simple linear regression was used to predict the correlation between the number of spiked cancer cells and their recovery. Analyses of the other data were performed by using a Student’s paired *t*-test. Data were presented as mean ± standard error of the mean (SEM), and *p* < 0.05 was considered statistically significant.

## 3. Results

### 3.1. The EpCAM^–^CD45^–^ Cells That Can Not Be Removed by PM Were Defined as Apoptotic Cells

**Figure 2:**
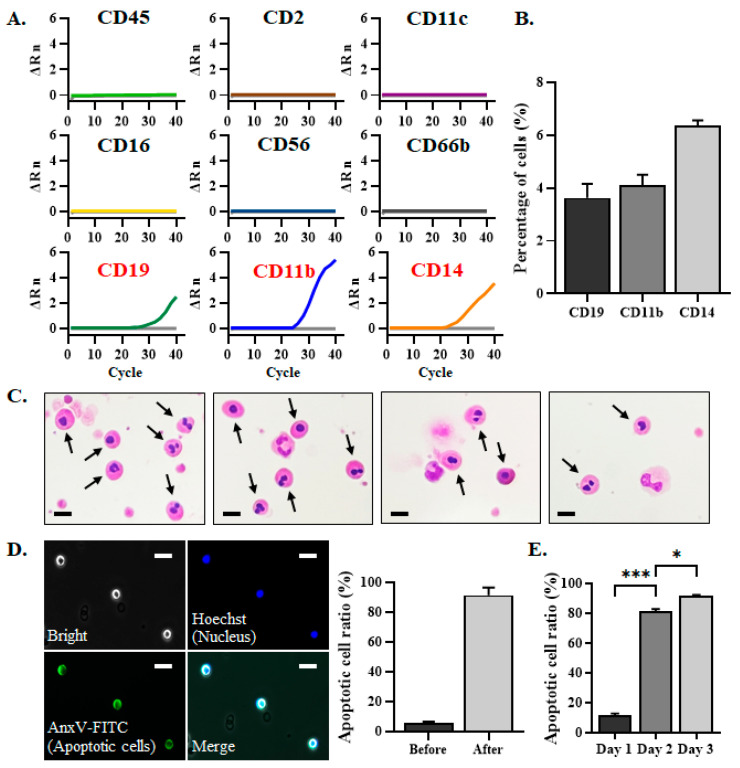
EpCAM^–^CD45^–^ cells present in the cell filtrate after PM I were mainly the apoptotic cells. (**A**) Peripheral blood (2 mL) from healthy volunteers was subject to the workflow of PM I. The cell filtrates were analyzed by real-time RT-PCR to determine the levels of mRNA expression for the indicated CD markers. Representative amplification curves are shown. Color lines and light-gray lines represent the amplification curves for the indicated antigens and the no template control, respectively. The light-gray lines in the top six panels were not clearly visible due to the overlap with the color lines. (**B**) Cell filtrates after PM I were placed on the slide for immunofluorescence staining using PE-conjugated antibodies against CD11b, CD14, and CD19, respectively. Fluorescence microscopy was used to define the percentage of cells that were positive for the indicated surface antigens. Data represent the mean ± SEM (n = 3). (**C**) Cell filtrates after PM I were cytospun on the slide, and Liu’s stain was performed for analysis of cell morphology using phase contrast microscopy. Apoptotic cells are marked with arrows. Scale bar: 10 μm. (**D**) Cell filtrates before and after the workflow of PM I were incubated with AnxV-FITC and Hoechst 33342 nucleus-staining dye followed by fluorescence microscopy analysis. The cell images illustrating the apoptotic cells (AnxV-FITC positive) are shown. Scale bar: 25 μm. Data represent the mean ± SEM (n = 3) for the ratio of apoptotic cells. (**E**) Peripheral blood collected from healthy volunteers was kept at 4 °C for 1–3 days and processed by PM I. The cell filtrates were incubated with AnxV-FITC for fluorescence microscopy analysis. Data represent the mean ± SEM (n = 3) for the ratio of apoptotic cells. *, *p* < 0.05; ***, *p* < 0.0001.

In addition to EpCAM^+^CD45^–^ CTCs, large numbers of EpCAM^–^CD45^–^ cells were present in the cell filtrates after depletion of the CD45^+^-leukocytes by PM I, regardless of the blood samples being from healthy donors or cancer patients [16]. To define the origin of the EpCAM^–^CD45^–^ cells, peripheral blood from healthy donors was collected and was subject to PM I to deplete CD45^+^-leukocytes after storage of the blood for 24 h. The remaining cells in the cell filtrates were analyzed by real-time RT-PCR for the expression of mRNA that corresponds to surface antigens representing leukocyte (CD45), T cells (CD2), dendritic cells (CD11c), NK cells (CD16 and CD56), granulocytes (CD66b), B cells (CD19), and monocytes and macrophages (CD11b and CD14), respectively (Figure 2A). Only the mRNA corresponding to CD19, CD11b, and CD14 were detectable in the cell filtrates obtained by PM I. Immunofluorescence staining revealed that only a small fraction of the cells expressed CD19 (3.6 ± 0.9%), CD11b (4.1 ± 0.7%), and CD14 (6.4 ± 0.3%), respectively (Figure 2B). Liu’s stain analysis revealed that most of the cells displayed the characteristic morphology of an apoptotic body, with condensed and rounded nuclei (Figure 2C). The cells with the morphology of an apoptotic body have also been observed in the cell filtrates after the depletion of CD45^+^-leukocytes in cancer patients’ blood samples by PM I. The AnxV-FITC binding assay further confirmed that most of the cells in the cell filtrates after PM I were apoptotic cells (Figure 2D). With about 10^7^ CD45^+^-leukocytes in a 2 mL blood sample, the ratio of apoptotic cells was 5.6 ± 1.9% before processing the sample by PM I (Figure 2D). These data indicate that apoptotic cells already exist in the blood sample. The number of CD45^+^-leukocytes was reduced to 10^3^~10^4^ after PM I, resulting in an increase in the ratio of apoptotic cells to 91.4 ± 8.9% in the cell filtrates after PM column (Figure 2D). These data indicate that apoptotic cells cannot be removed by PM I. The EpCAM^–^CD45^–^ cells, therefore, represent a pool of apoptotic cells that remained in the cell filtrates during CTCs isolation by the workflow of PM I.

In the clinical setting, CTC isolation and enumeration were usually performed on day 2 after blood collection. To determine whether the appearance of apoptotic cells was related to the storage of blood samples, the peripheral blood from healthy donors was subject to PM I on the day of blood collection (day 1), at 24 h (day 2), or at 48 h (day 3) after blood collection. The ratio of apoptotic cells in the cell filtrate after PM I was determined by AnxV-FITC binding, followed by fluorescence microscopy. Freshly prepared blood samples had a minimal ratio of apoptotic cells in the cell filtrates after PM I (Figure 2E). Apoptotic cells accounted for 81.4 ± 1.4% and 91.7 ± 0.5% of the cells that were present in the cell filtrates after PM I for the blood samples processed on day 2 and day 3, respectively. These cells were CD45^−^ and were not depleted by the anti-CD45 magnetic beads. While it may not be feasible to perform CTC isolation by PM I on the day of blood collection in a clinical setting, it is worthwhile to develop a strategy to deplete these EpCAM^–^CD45^–^ apoptotic cells to improve the purity of CTCs.

### 3.2. Preparation of In-House AnxV Beads for Binding and Elimination of Apoptotic Cells

**Figure 3:**
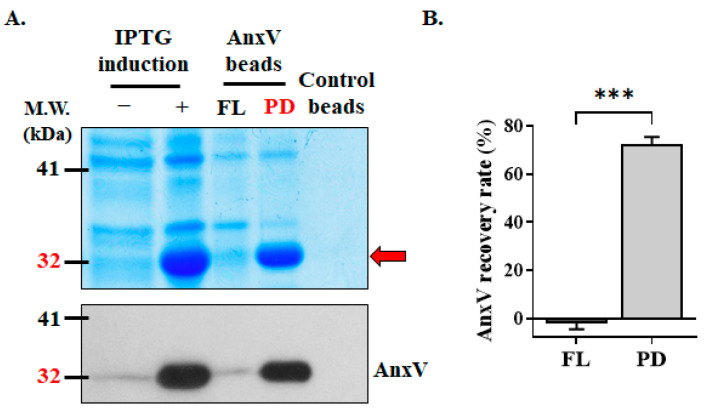
Preparation of in-house AnxV beads. (**A**) A recombinant *E. coli* bacterial strain carrying the plasmid (pET-14b-AnxV) encoding His-AnxV protein was induced by IPTG. In-house AnxV-conjugated beads were prepared as described in the Materials and Methods section. AnxV expression of the bacteria lysate before (−) and after (+) IPTG induction, as well as the presence of His-AnxV in the eluates (flow-through, FL) and on-bead (pulldown, PD), was characterized by Coomassie blue staining and Western blot analysis using the antibody against AnxV. (**B**) His-AnxV proteins which presented in the eluates (flowthrough, FL) or bound to the beads (pulldown, PD) were quantified. Data represent the mean ± SEM for the percentage of AnxV protein in FL and PD relative to the AnxV protein in the crude bacterial lysates (n = 5). ***, *p* < 0.0001.

In-house AnxV magnetic beads were prepared to remove the EpCAM^–^CD45^–^ apoptotic cells and to improve the purity of the CTCs obtained by PM I. His-tag AnxV proteins were purified from the bacteria transformed with the plasmid of pET-14b-AnxV after IPTG induction. AnxV was successfully induced by IPTG, as revealed by Coomassie blue staining and Western blot analysis (Figure 3A). After purification by using magnetic nickel beads, the AnxV protein in the bacterial lysates was enriched. Most of the recombinant AnxV proteins bound to the beads (pull down, PD) and minimally presented in the eluate (flow-through, FL), equivalent to a bead conjugation efficiency of 72.2 ± 3.1% (Figure 3B).

**Figure 4:**
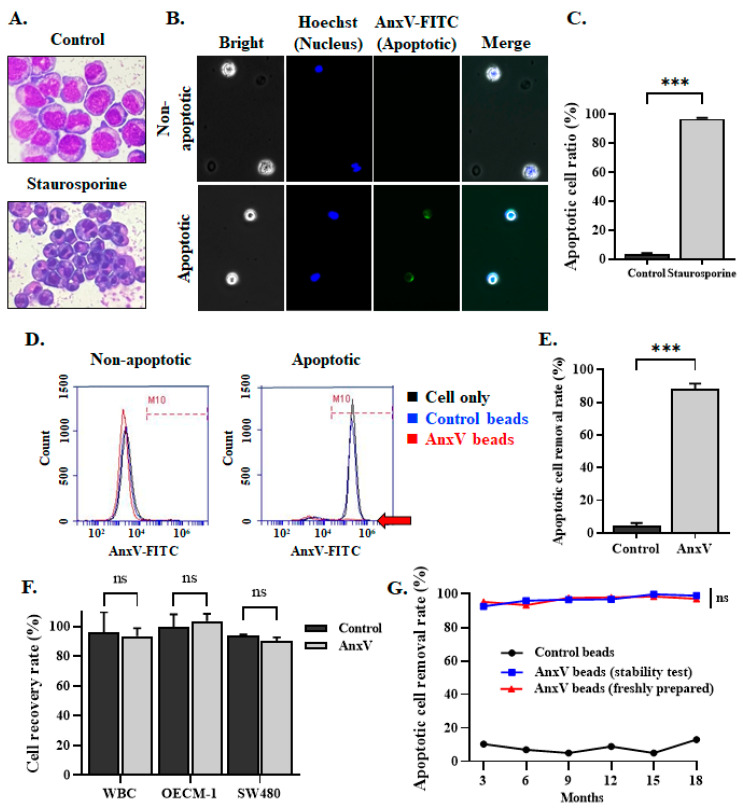
Evaluation of the in-house AnxV beads in the binding and removal of apoptotic cells. (**A**–**C**) Jurkat cells were treated with staurosporine to induce apoptosis. Cell morphology was observed by Liu’s stain (panel **A**). Images of the control non-apoptotic cells and apoptotic cells after staining with AnxV-FITC are shown (panel **B**). The ratio of apoptotic cells was determined by counting the number of cells that were AnxVpositive or negative (panel **C**). Data represent the mean ± SEM (n = 6). ***, *p* < 0.0001. (**D**,**E**) The non-apoptotic and apoptotic Jurkat cells were pre-incubated with the control beads or AnxV beads. Cell only without beads was used as a negative control. The cell suspension was collected and incubated with AnxV-FITC, followed by flow cytometry analysis. Representative histograms are shown in (panel **D**). The red arrow points to the histogram corresponding to the AnxV beads. The removal rate of the apoptotic cells was calculated and presented in panel **E**. Data represent the mean ± SEM (n = 8). ***, *p* < 0.0001. (**F**) The control beads or the AnxV beads were incubated with the non-apoptotic WBCs or the cancer cells (OECM-1 and SW480). The ratio of cells retained in the cell suspension relative to the input cell number was defined as the cell recovery rate. Data represent the mean ± SEM (n = 3). ns, not significant. (**G**) The efficacy of the AnxV beads in the removal of apoptotic cells was periodically analyzed up to 18 months after preparation and compared with the freshly prepared beads. The control beads were used as a negative control. Data on the apoptotic cell removal rate are plotted. ns, not significant.

To determine the efficiency of the in-house AnxV beads in the removal of apoptotic cells, a protocol for the induction of Jurkat cell apoptosis by treatment with staurosporine was established. Jurkat cells treated with staurosporine for 3 h displayed a characteristic apoptotic morphology, with condensed and rounded nuclei forming the apoptotic bodies (Figure 4A). Apoptotic, but not non-apoptotic cells, can bind commercially available AnxV-FITC and appear green under fluorescence microscopy (Figure 4B). Flow cytometry analysis revealed that only 3.3 ± 0.9% of the Jurkat cells without treatment of staurosporine-bound AnxV-FITC, while 96.6 ± 0.7% of the Jurkat cells treated with staurosporine underwent apoptosis and displayed AnxV-FITC binding (Figure 4C). These cells were, therefore, used as the control apoptotic cells in the following experiments.

For determining the capability and specificity of the in-house AnxV beads in the removal of apoptotic cells, Jurkat cells with or without treatment of staurosporine were incubated with the in-house AnxV beads or the control beads. The unbound cells in the supernatant were collected and subject to flow cytometry analysis using AnxV-FITC. In-house AnxV beads, but not the control beads, efficiently removed the apoptotic cells, with a removal rate equivalent to 88.3 ± 3.2% (Figure 4D,E). The in-house AnxV beads did not elicit any effect on non-apoptotic cells, regardless if the cells were Jurkat cells, WBCs, OECM-1 oral cancer cells, or SW480 colorectal cancer cells (Figure 4D,F), indicating the specificity and efficacy of in-house AnxV beads in the removal of apoptotic cells. The AnxV beads were stable even after storage for 18 months. The apoptotic cell removal rate remained unchanged when compared to the freshly prepared beads (Figure 4G). The in-house AnxV proved effective at binding and eliminating apoptotic cells.

### 3.3. Integration of AnxV Beads into PM Effectively Depletes the Apoptotic Cells in the Cell Filtrates

**Figure 5:**
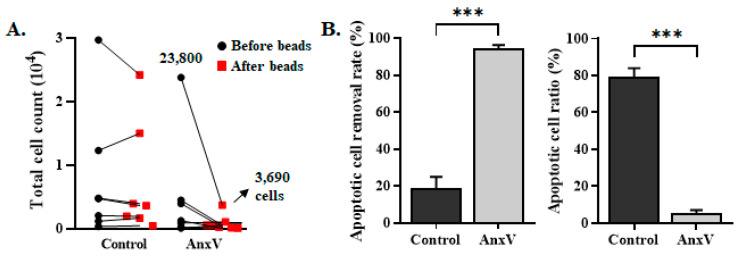
PM II effectively depletes the apoptotic cells in the cell filtrates. (**A**,**B**) CD45^+^-leukocytes of the peripheral blood from healthy donors were depleted by PM I. The remaining cell suspension was incubated with the control beads or the AnxV beads. The cells that were present in the suspension were counted and plotted (panel **A**). The residual cells in the final cell filtrates were stained with AnxV-FITC and Hoechst fluorescent dyes, followed by observation under fluorescence microscopy. The ratio of apoptotic cells (AnxV-FITC^+^ cells) was determined (panel **B** right), and the apoptotic cell removal rate was calculated (panel **B** left). Data represent the mean ± SEM (n = 7). ***, *p* < 0.0001.

The workflow of PM II was developed by integrating and placing AnxV beads after the workflow of PM I [16], with an aim to remove apoptotic cells and improve the purity of viable CTCs (Figure 1). The experiments were first performed to investigate the performance of PM II in the removal of the apoptotic cells that are present in the blood samples stored for 24 h. The cell filtrates, after the workflow of PM I, were incubated with either the control beads or the AnxV beads (Figure 5A). The control beads did not further reduce the number of cells present in the cell filtrate (before beads vs. after beads in the control bead group). In contrast, a large portion of the cells was removed by AnxV beads (before beads vs. after beads in the AnxV bead group). The total cell count in the cell filtrate was reduced to less than 1000 cells in six out of the seven samples, resulting in a significant decrease in the background apoptotic cells. The cell number was significantly reduced to 3,690 cells by use of the AnxV beads, even for the blood sample with a high cell count of 23,800. As determined by the AnxV-FITC binding assay, more than 94.6 ± 1.7% of the apoptotic cells were removed by the AnxV beads (Figure 5B). The ratio of apoptotic cells present in the cell filtrates was 79.3 ± 4.7% and 5.4 ± 1.7% for the control beads and AnxV beads, respectively (Figure 5B).

**Figure 6:**
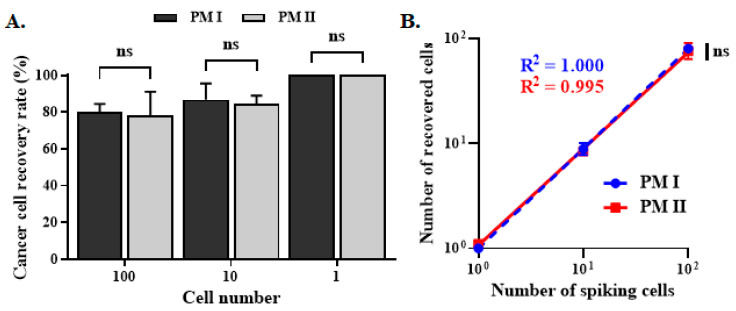
CTCs recovery rate for PM I and PM II. (**A**,**B**) The indicated amount of SW480 cells pre-labeled with Calcein AM red viability dye was spiked into the peripheral blood obtained from the healthy donors to mimic cancer patient blood samples. After processing through the workflow of PM I and PM II, the number of SW480 cells remaining in the final cell filtrates was counted. The cancer cell recovery rate was determined and plotted (panel **A**). The correlation of the number of spiking cells vs. the number of recovery cells was plotted, and the correlation coefficient was determined (panel **B**). Data represent the mean ± SEM (n = 3). ns, not significant.

SW480 cancer cells pre-labeled with Calcein AM red were spiked into the peripheral blood of healthy volunteers, creating a model that mimics cancer patients with CTCs in the peripheral blood. The mimetic samples were then subject to PM I and PM II for the removal of background cells. The recovery of cancer cells was similar for PM I and PM II. As low as one cancer cell can be detected. The correlation coefficient (R^2^) was equivalent to 1.000 and 0.995 for PM I and PM II, respectively (Figure 6A,B). PM II (PM I plus integration of AnxV beads), therefore, significantly decreases the background apoptotic cells, with no impact on the recovery of cancer cells.

### 3.4. CTC Enumeration and Mutant Gene Detection of Clinical Specimens with PM II

**Figure 7:**
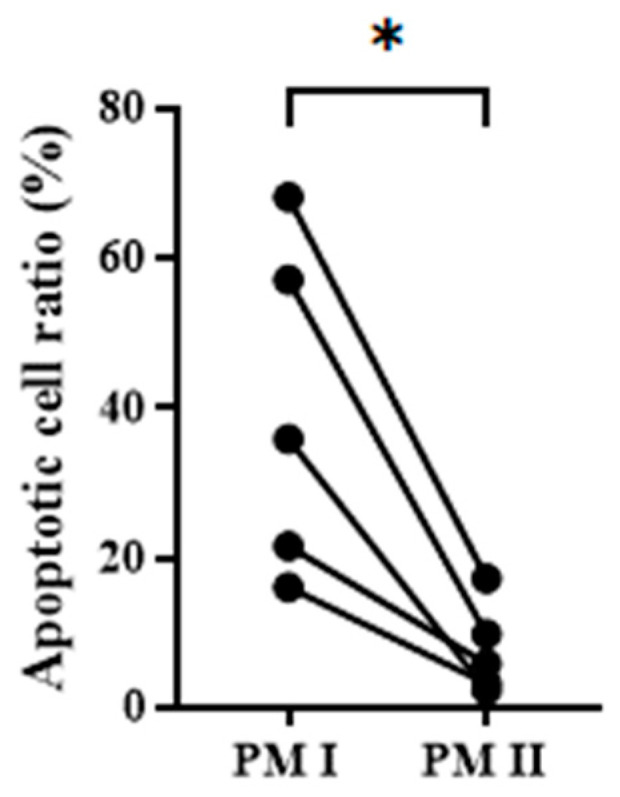
The ratio of apoptotic cells in the cell filtrates obtained by the workflow of PM I and PM II. Peripheral blood from patients with colorectal cancer (n = 5) was subject to the workflow of PM I and PM II. The residual cells in the final cell filtrates were stained with AnxV-FITC and Hoechst fluorescent dyes followed by observation under fluorescence microscopy. The ratio of apoptotic cells (AnxV-FITC^+^ cells) was determined and the paired PM I and PM II data from the same patient are plotted. *, *p* < 0.05.

To demonstrate the effectiveness of PM II for CTC detection in a clinical setting, peripheral blood from patients with colorectal cancer (n = 5) was collected and subject to the workflow of PM I and PM II, respectively. The number of apoptotic cells was significantly reduced by PM II. As a result, the ratio of background apoptotic cells ranged from 16.0% to 68.1% for PM I and from 3.2% to 17.2% for PM II, respectively (Figure 7). PM II, therefore, is effective in the removal of apoptotic cells from the cell filtrates.

Additional experiments were performed to compare the purity and number of CTCs collected from the peripheral blood of patients with colorectal cancer (n = 9) by PM I and PM II. The data indicate that the purity of CTCs was significantly increased by PM II. Despite a decrease in CTCs count for four of the nine samples, the total number of cells present in the cell filtrate was significantly reduced, resulting in an increase in the CTC purity (Table 1).

**Figure 8:**
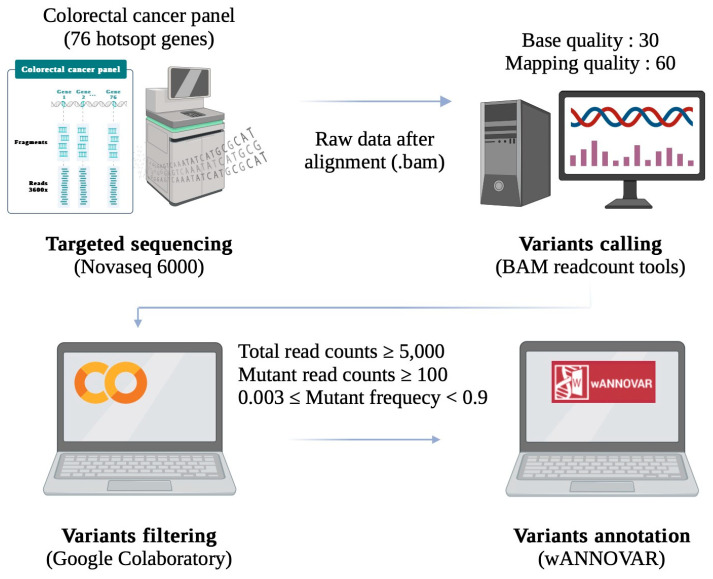
Workflow and methods of bioinformatics analysis.

Sufficient CTC purity is required for the detection of gene mutations by NGS. With the increase in CTC purity by PM II, we investigated whether or not PM II increased the confidence for detecting the gene mutations associated with CTCs. The cell filtrates obtained after the workflow of PM I and PM II were subject to WGA to amplify the whole genome of the CTCs and background cells. DNA amplified by WGA was analyzed by NGS using a colorectal cancer panel covering 76 genes, of which mutations were commonly found in patients with colorectal cancer. A computational workflow was established for bioinformatic analysis of the data (Figure 8), which included the alignment of reads to the reference genome sequences to generate the BAM file, variant calling by the BAM readcount tools, variant filtering by using Google Colaboratory, and variant annotation by wANNOVAR.

Mutation at a specific gene position was considered significant when the read count for the mutation was ≥100. Only the pathogenic mutation of the MSH2 gene at *c.942+2T>G* (read count = 724) was considered significant for sample 1 when the cell filtrates obtained after the workflow of PM I were analyzed (Table 2). Seven pathogenic mutations at the MUTYH, MSH2, MSH6, and PTPN12 genes were considered significant when the cell filtrates obtained after the workflow of PM II were analyzed. The read counts were increased by 30.8–1122% when PM II was compared to PM I. For sample 2 and sample 3, only one mutation at the MSH2 gene was detected for the cell filtrates obtained from PM I and PM II. The read count for the mutation of sample 2 was increased from 249 for PM I to 450 for PM II, representing an 80.7% increase in the read count. The read count for the mutation of sample 3 was increased from 166 for PM I to 212 for PM II, representing a 27.7% increase in the read count. For sample 4, only one mutation at the MSH6 gene was detected for the cell filtrates obtained from PM I and PM II. The read count for the mutation of sample 4 was increased from 357 for PM I to 557 for PM II, representing a 64.1% increase in the read count. The increased number of mutant reads lends confidence to the findings concerning the indicated gene mutations in the CTCs. The workflow of PM II, therefore, increases the CTC purity and facilitates the direct detection of gene mutations by NGS.

## 4. Discussion

There is tremendous potential for the clinical application of CTCs, including the early diagnosis of cancer, monitoring the effects of treatment, and determining prognosis [25,42,43,44,45,46]. Studying isolated CTCs aids in probing the biological insights related to cancer metastasis and in discovering novel cancer biomarkers. In the present study, apoptotic cells are defined as the major cell population present in the cell filtrates collected through the workflow of PM I for CTC isolation. Based on this, a new version of workflow, PM II, is established for isolating CTCs with improved purity by the efficient depletion of background apoptotic cells using AnxV beads. The increase in CTC purity allows for direct profiling of cancer-related gene mutations without additional CTC picking or purification.

CTCs have been enriched based on their biological or physical properties [9,10]. In addition to CTCs, a high percentage of non-CTC background cells are usually present in cell filtrates collected from CTC isolation devices. For example, CTCs isolated by CellSearch are usually accompanied by a few hundred to a few thousand non-CTCs in the final cell filtrates [47]. Similar findings have been found in other commercially available CTC isolation platforms [48,49,50,51]. Due to the intrinsic characteristics of negative selection, the platforms operated by the principle of negative selection usually have lower CTC purity when compared to platforms based on positive selection. This hampers its further clinical application, such as the direct profiling of cancer-related gene mutations. Various manual or automatic cell-picking devices and methods have been developed to resolve this issue [52,53,54,55]. However, the devices and consumables are expensive, and cell-picking and isolation processing are usually time-consuming.

An investigation was initiated to characterize the cellular properties of the marker-negative EpCAM^–^CD45^–^ cells in the cell filtrates collected by PM I. Through the analyses performed in this and the previous studies [16], the EpCAM^–^CD45^–^ cells are typically heterogeneous, with some of the cells displaying unusually large size. Only a small portion of these cells is positive for CD146 (an endothelial cell marker), CD34 (a stem cell and endothelial progenitor cell marker), CK (an epithelial cell marker), CD19 (a B-cell marker), CD11b (a monocyte and macrophage marker), or CD14 (a monocyte and macrophage marker). These cells represent only a minor portion of the EpCAM^–^CD45^–^ cells. The origin of EpCAM^–^CD45^–^ cells may be heterogeneous, and they may not have originated from a single specific cell type. Further characterization of this cell population revealed that the EpCAM^–^CD45^–^ cells are mainly relative to the apoptotic cells. This is supported by the findings that large numbers of cells in the cell filtrates display the characteristic morphology of apoptotic bodies, with rounded nuclei and condensed chromatin. In addition, most of these cells bound AnxV, another property of apoptotic cells. Most of the background cells (i.e., EpCAM^–^CD45^–^) were removed by in-house-generated AnxV beads. As supported by the following findings, the EpCAM^–^CD45^–^ cells are likely derived from the CD45^+^- or CD45^–^ leukocytes undergoing apoptosis. First, EpCAM^–^CD45^–^ cells are present in the peripheral blood of healthy donors and cancer patients. Second, storage of the blood samples for a longer period usually accompanies an increase in the number of EpCAM^–^CD45^–^ nucleated cells. When CD45^+^-leukocytes undergo apoptosis, the expression of CD45 is weakened [56]. A change in antigen levels occurs with apoptotic neutrophils [57,58]. The current study, therefore, contributes to our understanding of the cellular properties of the EpCAM^–^CD45^–^ cells present in the cell filtrates obtained by the workflow of PM I for CTC isolation.

According to the cellular characteristics of the EpCAM^–^CD45^–^ cells, PM II was developed to isolate CTCs with improved purity. The AnxV beads were integrated into PM I for the removal of apoptotic cells. The performance of PM II in the enrichment of CTCs is similar to PM I. Despite the fact that the system could not surely detect one cancer cell, it can effectively remove the background cells. CTC enumeration can be more efficient and accurate and is less labor-intensive when using PM II. Profiling of cancer-related gene mutations can be performed without further purification and cell picking and can be more informative, as demonstrated by the sequencing data obtained from the four patients with colorectal cancer. The number of read counts was increased significantly for the CTCs obtained by PM II when compared to PM I. Gene mutations of MUTYH, MSH2, MSH6, and PTPN12 in CTCs were identified in four patients with colorectal cancer. Mutations in the base excision repair (MUTYH) and the mismatch repair (MSH2 and MSH6) genes have been associated with early-onset colorectal cancer [59]. A variant in PTPN12 can increase the risk of colorectal cancer [60]. Our study, with a limited number of clinical cases, provides a preliminary work for proof of principle and indicates that PM II is a suitable platform for CTC enumeration and mutation detection in a clinical setting. Large-scale and comprehensive clinical studies may further provide a definitive conclusion. A higher purity of CTCs obtained by PM II should also facilitate other CTC downstream applications, such as proteomic, transcriptomic, and drug screening analysis [61].

Despite PM I and PM II presenting the same detection limit of one cancer cell, the number of cancer patient CTCs collected by PM II is lower than by PM I. Previous studies indicate that apoptotic CTCs are present in the peripheral blood of cancer patients [62,63]. CTCs may also undergo apoptosis during storage of a blood sample [64] and form EpCAM^–^CD45^–^ cells. The use of AnxV beads to remove background EpCAM^–^CD45^–^ cells may, at the same time, eliminate apoptotic CTCs and have an impact on the recovery of CTCs. While the incidence of apoptotic CTCs is higher in patients with breast cancer at an early stage, compared to those with metastasis [65], and the proportion of CTCs with features of early apoptosis is a prognostic indicator of metastasis-free survival and a neoadjuvant chemotherapy response for patients with breast cancer [66], it is not clear whether the decrease in the total CTC count due to removal of apoptotic CTCs has any clinical impact. In our previous studies, we noted that the changes in the CTC count during the follow-up of patients are more important than the absolute CTC count. For example, blood samples collected at baseline and two weeks after baseline from the patient with head and neck squamous cell carcinoma were compared. A decrease in the CTC counts indicates an effective chemotherapy [19]. Sequential CTC enumeration during treatment can supplement standard medical tests to unveil whether patients with locally advanced or metastatic HCC, particularly for the AFP-low cases, have a stable disease, partial response, or disease progression [25]. Hence, the removal of apoptotic CTCs and a lower CTC collection by PM II may not have an impact on the correlation of CTC count with clinical outcomes, such as prognosis and treatment response. It is clear from our study that, by removing the EpCAM^–^CD45^–^ cells, a higher purity of CTCs can be obtained, leading to better coverage for mutation detection by NGS. It is warranted to address this issue further by enrolling patients in clinical studies and delineating whether the improvement of purity and the lower recovery efficiency of CTCs by PM II have any impact on its clinical applications for cancer patients when compared to PM I.

## 5. Conclusions

By defining the major population of EpCAM^–^CD45^–^ cells as apoptotic cells, AnxV beads were developed in-house and integrated into the workflow of the PM I CTCs isolation platform to form a new platform of PM II. It holds great promise as a platform for leukocyte depletion, CTC enumeration, and direct molecular characterization and analysis of CTC genetic mutations in a clinical setting.

## Figures and Tables

**Table 1:** Improvement of CTCs purity by PM II.

Sample	PM I			PM II			Improvement of Purity ^a^
	Total Cell Number	CTCs Count	Purity	Total Cell Number	Ctcs Count	Purity	
1	49,623	2	0.004%	3739	4	0.107%	2575%
2	4338	7	0.16%	610	4	0.66%	313%
3	20,985	8	0.04%	4390	3	0.07%	75%
4	29,706	7	0.02%	4531	3	0.07%	250%
5	60,210	14	0.023%	20,084	7	0.035%	52%
6	46,168	0	0.00%	3081	1	0.032%	NA ^b^
7	32,622	0	0.00%	6546	1	0.015%	NA
8	17,641	0	0.00%	6550	0	0.00%	NA
9	6561	0	0.00%	1553	0	0.00%	NA

^a^ Improvement in purity was calculated using the equation: purity for PM II—purity for PM I/purity for PM I.; ^b^ NA: not available.

**Table 2:** The pathogenic positions and sequencing outcomes of four specimens obtained after PM I and PM II.

	ClinVar Database Colorectal-Related “Pathogenic Position”			Read Counts		
Sample	Gene Name	Gene Alternation	Protein Alternation	PM I	PM II	Improvement ^a^
1	*MUTYH*	*c.1103-2A>G*	-	13	100	669%
		*c.548G>A*	p.Gly183Asp	26	107	312%
		*c.544C>T*	p.Arg182Cys	9	110	1122%
		*c.452A>G*	p.Tyr151Cys	37	160	332%
	*MSH2*	*c.942+2T>G*	p.Leu310Pro	724	942	30.8%
	*MSH6*	*c.718C>T*	p.Arg240Ter	78	134	71.8%
	*PTPN12*	*c.182A>G*	p.Lys61Arg	72	109	51.4%
2	*MSH2*	*c.942+2T>G*	p.Leu310Pro	249	450	80.7%
3	*MSH2*	*c.942+2T>G*	p.Leu310Pro	166	212	27.7%
4	*MSH6*	*c.718C>T*	p.Arg240Ter	357	557	64.1%

^a^ Improvement was calculated using the equation: read counts for PM II—read counts for PM I/read counts for PM I.

## Data Availability

All relevant data are included in the paper.
